# Barriers and Access to Care for Firefighters With Posttraumatic Stress Disorder Seeking Ketamine-Assisted Therapy: A Qualitative Study

**DOI:** 10.1097/JOM.0000000000003665

**Published:** 2026-02-02

**Authors:** Vivian W.L. Tsang, Tavneet Walia, Katherine Sattler, Pamela Kryskow

**Affiliations:** From the Department of Psychiatry, Faculty of Medicine, University of British Columbia, Vancouver, Canada (V.W.L.T.); Faculty of Science, McMaster University, Hamilton, Canada (T.W.); Faculty of Science, University of British Columbia, Vancouver, Canada (K.S.); and Roots to Thrive Nonprofit Society, Vancouver, Canada (P.K.).

**Keywords:** ketamine-assisted therapy, treatment-resistant PTSD, firefighter mental health, psychedelic-assisted therapy, barriers to treatment, mental health stigma

## Abstract

**Objective:**

Firefighters are exposed to a disproportionately high number of traumatic incidents and thus display elevated rates of trauma-related mental health disorders, with the reported average PTSD prevalence being 7.3% in firefighters versus 1.3%–3.5% in the general population. Oftentimes, conventional treatments demonstrate limited efficacy. Ketamine-assisted therapy (KAT) has recently arisen with promising long-term results.

**Methods:**

The experiences of six firefighters who had enrolled in but not begun the Roots to Thrive (RTT) KAT program were investigated. Interviews were conducted pretreatment, and a thorough thematic analysis was performed.

**Results:**

Four major themes were identified: (1) feeling stuck and reaching a breaking point, (2) stigma surrounding PTSD and the use of ketamine for treatment, (3) the importance of self-advocacy in navigating access to treatment, and (4) substantial financial and logistical barriers.

**Conclusions:**

This study highlights the importance of system-wide changes necessary to support treatment-seeking individuals.

## Abstract 2

Conventional treatments have displayed limited long-term efficacy for firefighters with treatment-resistant mental health disorders, leading to significant negative implications on their day-to-day lives. Ketamine-assisted therapy (KAT) has emerged as an alternative to traditional treatment methods, although significant stigma exists in the use of psychedelic treatments, causing deterrence.

## Graphical abstract

**Figure:**
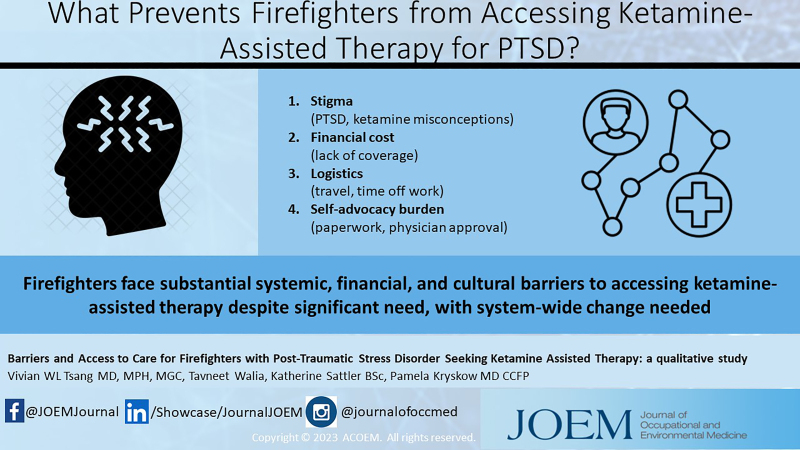

**LEARNING OUTCOMES**

- Understand the pervasiveness of PTSD among firefighters and the lack of success they have found in other treatments they have utilized
- Analyze the four major themes that emerged from firefighter interviews that describe the experiences of the firefighters in order to access ketamine-assisted therapy (KAT)

Firefighters face routine occupational risks and are often put in high-stress, high-consequence environments. Accordingly, 96.4% are exposed to a potentially traumatizing event, and 90% report responding to an event involving a fatality at some point throughout their career.^1^ Previous studies have displayed that there are likely long-term consequences of repeated exposure to traumatic events (RET) in firefighters, and this is closely related to other mental health outcomes and their overall general well-being.^2^ Other studies have demonstrated the correlations between event load (duty-related potentially traumatic events) and PTSD symptoms.^3^ This high rate of exposure is accompanied by high rates of trauma-related disorders among firefighters.^4^ Recent estimates put the prevalence of PTSD at 14.3% compared to the yearly rate of 3.5% in the general Canadian population.^5,6^ Similarly, review papers put their prevalence of depression at 18.7%, whereas the rates of life alcohol use disorder (AUD) and anxiety are as high as 50% and 12%, respectively.^4,7^ These rates all fall above the prevalence in the general population.^8,9^

First-line treatments of TRMI remain limited. In the case of depression, it is estimated that 30% fail to respond to treatment. Antidepressants have been associated with unwanted side effects, and both medication and psychotherapy suffer from high rates of nonresponse and premature discontinuation 9–11. For PTSD, Cognitive Processing therapy (CPT) is strongly recommended as a first-line treatment by the American Psychiatric Association.^10^ However, dropout rates are 47% in one study and the nonresponse rate for CPT is as high as 50%.^11^ Dropout rates for other modalities such as prolonged exposure (PE) are also high at over 55% in one study. Furthermore, patients with PTSD who receive treatment in a primary care setting have a 30% rate of recurrence.^12^ Similarly, findings in veteran populations show that 69% relapse after treatment of AUD.^13^ There are also sleep-specific treatments that can be highly beneficial in this population, including CBT for insomnia, exposure, and relaxation and rescripting therapy for addressing trauma-related nightmares.^11^ Despite treatment, repeat trauma exposures and life stressors can often worsen PTSD symptoms.^11^

Ketamine-assisted therapy (KAT) has been used historically for the treatment of mental health disorders such depression and, in more recent years, gained notoriety for treating PTSD. A recent study found that KAT was highly tolerable and safe with no dropouts in the trial.^1^ In treating depression and anxiety, KAT has proved effective and improved the well-being of two-thirds of patients with problematic substance use.^14^ Likewise, meaningful improvements have also been found in studies for the treatment of PTSD with KAT.^15,16^

Currently, the drug ketamine is the only psychedelic drug that is widely available for legal access in Canada. It is available through controlled distribution at approved clinics and must be administered by a medical professional at designated clinics. Roots to Thrive (RTT) is Canada's first and only multidisciplinary, nonprofit health care practice to legally offer multiweek, group therapy KAT programs. They use a community of practice model, uniquely designed to address trauma and to promote resilience. RTT offers sessions specific to firefighters, which are carried out by the RTT team members who have been trained to facilitate psychedelic therapy.

Despite the potential of KAT to treat TRMIs, there are several potential obstacles that may limit its access. As KAT is not yet covered by the BC Medical Services Plan, the cost of the treatment must be covered by other means. Additionally, a patient must also have a prescription from a medical doctor in order to receive KAT. Patients in the general population with depression and substance use disorders also report feeling that the use of ketamine is highly stigmatized. However, to date, no studies have looked into barriers, facilitators, and the process of accessing KAT for firefighters specifically. Understanding the path to treatment for this population is critical given the high prevalence of mental health disorders within the firefighting profession and the association with occupational risks.

## METHODS

In this study, six participants were recruited. The firefighters who were invited to participate in the study were diagnosed with treatment-resistant mental health disorders and have enrolled in but not begun the 12-week Roots to Thrive Ketamine–assisted therapy (RTT-KAT) program. Screening was done preliminarily to assess participant eligibility, and the risks of the study were explained to the participants in lay terms by a research team member. Once the participants provided consent, they were assigned a unique study ID number and received a copy of the original signed consent form. Ethics proposals were approved by Veritas IRB on July 17, 2024 (IRB tracking number 2024-3569-18737-5).

### Recruitment and Eligibility

Mental health disorders were evaluated and diagnosed by primary care physician or psychiatrist referral, and intake assessment was completed by a psychiatric RN or by an MD (medical intake). The eligibility criteria of the participants who were planning on participating in the study (thus, have planned to participate in the RTT-KAT program) were as follows: 19 years or older, must be a firefighter of any working status (full or part time, retired, on leave or volunteer), referred to RTT-KAT by a longitudinal primary care provider, and diagnosed with a mental health disorder (including depression, chronic anxiety, OCD, PTSD, unresolved grief, adjustment, or acute stress disorder). They must also have attempted at least one other treatment option unsuccessfully, understand the consent form/study procedures/agree to participate, and must be a fluent English speaker. Additionally, the investigator of the study had the right to deem a potential participant fit or unfit to participate in the study.

The exclusion criteria were primarily based on RTT-KAT ineligibility, which included dementia or delirium diagnosis, active psychotic symptoms, high risk of coronary artery disease, uncontrolled cardiopulmonary disease/cardiovascular disease or hypertension, current aneurysm, intracerebral hemorrhage/hepatic cirrhosis/hepatorenal disease history, possibility of suspected pregnancy, or planning to become pregnant during the course of the study.^17^ Overall, other major concurrent illnesses that are clinically significant and may interfere with study results would cause the exclusion of a participant.

Through a review of literature, existing surveys, and opinions, a two-page demographic and substance use questionnaire was developed and administered to the participants prior to the conduct of individual interviews. The interviews displayed the participants' mental health struggles triggered by their jobs as firefighters and workplace environments, their previous attempts at treatments, and the process of getting to RTT-KAT. The interviews were recorded, and transcripts were created to analyze the responses further.

### Data Analysis

The quantitative data collected from this study have been reported elsewhere.^18^ In a preliminary coding process, data from the interview responses were reviewed individually by two independent reviewers to create general themes (Supplementary data in Appendix 1, http://links.lww.com/JOM/C366). The preliminary themes were reviewed by a third, senior reviewer. These initial themes were revised to create the main themes of focus for the study, with relevant subthemes and participant responses grouped together. Then, the independent reviewers coded all the relevant data, with inconsistencies managed by the third senior reviewer.

## RESULTS

### Demographic Data

A total of eight participants (one female) were enrolled to complete the 12-week RRT-KAT program; six completed interviews.^19,20^ One participant identified themselves as indigenous. These details are not reported in the table to preserve patient confidentiality. Three participants listed PTSD as their primary diagnosis, and three listed PTSD as their secondary diagnosis. Two participants listed GAD and TRD as their primary diagnosis.

#### Feeling Stuck With Previous Treatment and Reaching a Breaking Point

Throughout the interviews, each of the participants detailed their journey with trauma and mental health disorders before KAT (Table 1). A common sentiment expressed was that they could not keep coping anymore. The severe effects of mental health disorders included emotional numbness, night terrors, and extreme anxiety. In many instances, they added that they were no longer able to suppress these symptoms, which they felt were controlling their lives. Participants discussed a gradual build-up and burnout, as one explained, “You continually are always going to other trauma calls, so it kind of builds up layer, layer, layer after layer.” Another participant echoed this sentiment, stating, “I started getting increasingly worried more and more about doing that one call that would be a career-ender.”

**TABLE 1.** Summary of the Qualitative Data

Theme	Description	Exemplar Quotes
Stigma	Uninformed opinions and the impact of societal views	“There is a stigma around it even for someone like me, who's involved in mental health on a daily basis” “I definitely feel a stigma around having a PTSD claim” “People that are struggling with PTSD or haven't identified that they have it wouldn't think to look at this program…it's just hippie stuff, right?” “I could see how this stigma behind it could maybe hold back some people”
Self-advocacy and self-education	Taking the initiative to inform oneself about trauma and treatments. Making informed and assertive requests to WorkSafe case managers and physicians	“I had already been involved in learning about psychedelics for a number of years.” “I knew what I wanted to do and if she would have signed off, I just would have gone somewhere else.” “I was lucky that I was prepared to do the paperwork and even knowing, even with all the information I had, it was hard. It was draining and it was scary.”
Awareness about KAT	Disconnect between information about KAT as a treatment option and people who would benefit from accessing it	“There has to be media about it” “I think the biggest thing is making everybody aware” “I never would have thought of it, and until you know, you just reading about it and magazines and online and that sort of thing”
Feeling stuck with previous treatment	Other types of therapy (talk therapy, EMDR, CBT, etc) having limited efficacy or short-term effects	“I actually I was seen a couple different counselors, but just that it just I wasn't getting anywhere.” “We were trying to work through and some of the things were extremely difficult and not really getting a lot of progress”
Logistical barriers	Obstacles to accessing KAT including a lack of financial resources, difficulties getting time off, and getting to and from the sessions	“I would not have been able to do it financially if it wasn't covered by WCB.” “I can afford to throw that money at it if I need to. But you know, some other people can't…. it cost the hotel cost, the ferries, you know it adds up after a while”
Reaching a breaking point with mental health struggles	Long-term, compounding trauma leading to a crisis or severe effects on a patient's day-to-day life	“I didn't really have a I didn't really give myself much of a choice. I had to do something.” “I was going down the path of using alcohol in a bear running unhealthy way.” “I just needed to see change, I'd say like I was at a bottom with my whole mental health”

Many reported having a crisis or realization that they had to make a change:

“It was a pretty wild call. So after I was, I was instantly like … I can't keep going like this without at least some help from somebody.”

“If I want to call it a kind of an existential crisis thing that happened to me. And that's when I was like, OK, something's going on here. Something's wrong.”

A recurring theme in their experiences was trying many different counselors and treatment modalities for their mental health with little permanent success. The treatments mentioned included EMT, EMDR, talk therapy, and CBT, many of which were not first-line therapies for trauma. Feeling “stuck” and making minimal progress were reported on several occasions, as one participant put it: “I was stuck on a bit of a hamster wheel.” Other participants noted feeling better for “little short stints” and that “things aren't cured by any means, but things have gotten better”, following forms of therapy other than KAT.

#### Stigma

The word “stigma” was used by participants 12 times across all 6 interviews. Throughout the interviews, participants brought up the ways in which the use of ketamine and their mental health diagnosis were stigmatized.

They discussed how ketamine and stigma affected their personal relationships: some noted keeping their treatment a secret from family members, whereas one participant stated that their partner was a hurdle because “she thought I was gonna get addicted and I'm a druggie.” Participants also reported stigma as a barrier to accessing KAT; it was mentioned that people struggling with PTSD might not consider it an option because it is “hippie stuff.” For firefighters wanting to undergo KAT, a participant recounted that a physician refused to sign off on KAT treatment because “they didn't know anything about it and they didn't even educate themselves. They just said no.”

Regarding their mental health and diagnosis, participants touched on the culture among firefighters in the workplace. They spoke about firefighters having a “suck it up mentality,” and in the case of a meditative retreat, one participant said, “First responders are really good at saying, oh, that was stupid.” Some participants reported telling very few colleagues about their PTSD claims and mentioned that other firefighters would avoid them because of their diagnosis. However, they also noted improvements in this culture and an increase in conversations about mental health. According to one participant: “Around the coffee table and lunch table, more and more guys that I work with are like talking about it and being open … I don't think that was happening at all years ago.”

#### Self-Advocacy and Awareness

Participants talked about the effort that was required of them during the journey to accessing KAT. In response to traumatic events at work, one participant explained the lack of support, stating that the attitude of WorkSafe was “Basically, too bad. Suck it up. Move on so. At that point, it kind of didn't know where to go, so I just kind of looked for my own help.” Several participants spoke about spending years teaching themselves about psychedelics and mental health and explained that they undertook this in response to their declining mental health:

“I turned to education around trauma and how the brain works and why I was acting the way I was. It sort of was my way of self-therapy.”

Many quotes from the interviews show patients' willingness to overcome administrative burdens. These burdens include excess paperwork and having to advocate for themselves to get their doctors to sign off:

“I was lucky that I was prepared to do the paperwork, and even knowing, even with all the information I had, it was hard.

It was draining and it was scary.”

“He was really hesitant. And I actually asked him what he needed and if it would help if he spoke with the medical director.”

Their recommendation for other patients seeking the same treatment was to make an effort to reach out to other people who had been through the program. Beyond this, participants were concerned that people suffering were not getting the right information that KAT could be a treatment in the first place. Some admitted that they did not think they were eligible or that, initially, they did not consider it, as one participant explained: “I didn't talk to (my doctor) about the ketamine-assisted therapy. I didn't know about it at the time.” Getting the word out, getting information into the media, and increasing conversations among peers were recommended.

#### Logistical Barriers

Logistical barriers were mentioned by five out of six participants. These included barriers to physically commuting, paying for, and having time away for the KAT sessions.

Participants mentioned several complications that came with transportation to the treatment facility. They pointed out that, aside from the price of the treatment itself, paying for cabs and ferries adds to the cost.

Coordinating the timing of their travel was also noted as one participant spoke about the timing of the ferry ride home: “I got to wait in the ferry terminal order like 2 1/2 hours before I hop on an hour and a half ferry back to Horseshoe Bay and the whole time I'm coming down from ketamine.”

The length of time spent traveling and away from home was cited as an obstacle, as was taking time off from work. One participant noted that “The time was a thing… I just took the sick time. Before, I would have been afraid to.”

Participants pointed out that, without access to funding for this therapy, the cost is a significant barrier. One participant expressed frustration over the lack of willingness of departments to fund the entire costs and give ample time off.

## DISCUSSION

To our knowledge, this is the first study exploring firefighters' views on the process of accessing KAT. The results provide important insights into the conditions under which these six firefighters sought out KAT, the role of public views in this process, and the obstacles they faced. Overall, the interviews show that firefighters need to put in significant effort to access KAT treatment and that many systemic barriers must be tackled before KAT can become more widely available to this population.

The barriers experienced by these six firefighters closely resemble those reported while accessing other mental health care for firefighters and first responders, suggesting that challenges in accessing KAT are reflect broader systemic issues in mental health care infrastructure.^21,22^

The results show that, based on the experiences of these six firefighters, they seem to be turning to KAT after trauma has severely affected their lives for long periods. Often, the decision was triggered by a crisis or a feeling of reaching rock bottom, and many of the quotations convey feelings of desperation. Ketamine interest has been associated with desperation around mental health; however, given its efficacy, making KAT a treatment option earlier in the process could alleviate unnecessary suffering.^23^ The patterns observed in this study mirror results from other studies, where firefighters delay seeking care until symptoms became “big problems,” often becoming a barrier themselves, despite understanding the importance of intervention.^24^

Participants emphasized that there was a disconnect between those struggling with mental health disorders and knowledge about KAT. Specifically, awareness that KAT exists, its efficacy, and who is eligible need to be addressed, according to our findings. The gap in knowledge dissemination likely mirrors that of more classic psychedelics. It has been found that the public generally has a neutral perception of the clinical efficacy of psychedelics and is unaware of the medical avenues to access them.^7,25^ In studies with firefighters engaging with conventional therapy, they noted similar gaps in the understanding of mental health providers of the nature of firefighting. Some also noted problems not only in terms of effectiveness but also in their confidence in the value of the treatment options available.^24^

In addition to a lack of awareness, participants expressed how they felt ketamine was stigmatized. This stigma has, in the literature, often been attributed to the historical criminalization and politicization of psychedelic substances, which leads to the ostracization of patients who use these substances.^26,27^ The present study highlights how misconceptions and narrow-minded views of the drug are present at multiple levels: participants face internalized stigma and stigma from their loved ones and from their physicians.

The finding of self-stigma echoes the results found by Jilka et al,^28,29^ who reported that KAT-naive patients with treatment-resistant mental health conditions were concerned about the stigma around ketamine. Similarly, studies on psychologists have found that stigma plays a role in shaping their views around psychedelic compounds.^30,31^ The participants of our study overwhelmingly cited this as a barrier to patients accessing KAT. Given the promising role of KAT during the mental health epidemic, this public attitude must be changed to allow participants to feel more comfortable in opting to use psychedelic substances as treatment.^32^

In the literature, stigma has also been consistently identified as a major barrier to accessing mental health treatment among firefighters, which could indicate that ketamine-related stigma may compound an already well-established cultural reluctance to seek care for PTSD and other trauma-related disorders.^24^

Lastly, participants faced administrative burdens caused by bureaucratic processes coupled with the burden of having to travel to receive KAT treatment. Streamlining paperwork, having easier routes for physician approval, and more local KAT clinics should be considered to address these hurdles. Other studies indicate the overwhelming contribution logistical barriers have on access to care in PTSD patients and further exacerbate PTSD symptomology.^33^ A reduction in logistical barriers would alleviate some of this stress and allow for more PTSD-affected firefighters to opt for this treatment. Overall, minimizing administrative and logistical barriers may thus have benefits beyond KAT, improving access to trauma-focused mental health care for firefighters and first responders more generally.

## CONCLUSION

The four major themes synthesized in this small cohort of only six patients highlight the importance and need for further research and studies with larger cohorts to have overarching impacts on the study of PTSD and other mental health-related disorders that first responders suffer from. Overall, greater funding, awareness, and administrative support are needed for firefighters diagnosed with PTSD to successfully receive and undergo treatment.

## ACKNOWLEDGMENTS

We would like to acknowledge all the staff and patients at Roots to Thrive for their contribution to psychedelic research.

## Supplementary Material


