# Supplementary material for: Barriers and Access to Care for Firefighters With Posttraumatic Stress Disorder Seeking Ketamine-Assisted Therapy: A Qualitative Study
Source: J Occup Environ Med. 2026 Feb 2;68(7):e472–6. doi: 10.1097/JOM.0000000000003665 (PMC13290054; doi:10.1097/JOM.0000000000003665)
Supplement: Supplementary file 1 [file joem-68-e472-s001.docx]

**Supplementary data:**

| Theme | Description | Exemplar quotes | Results/Discussion |
| --- | --- | --- | --- |
| Stigma | Uninformed opinions and the impact of societal views. | P1: “So there is a stigma around it even for someone like me, who's involved in mental health on a daily basis”  “My mom doesn't know that I went and and did ketamine. So there is a stigma around it”  “Back then you would you would never file a work safe claim for mental health, injury.”  P2: “I didn't really talk about it at work too much…  There is not a lot of people that work that know I have a PTA, PS PTSD claim.”  “ I definitely feel a stigma around having a PTSD claim”  “as a woman I will of course you have PTSD claim because because well, you know, you've always been really caring me.You've always been really sensitive and you're a delicate flower.”  “there needs to be some understanding, habitation of the stigma that detached with.  With talking about this program before you go through the program.”  “People that are struggling with PTSD or haven't identified that they have it wouldn't think to look at this program…it's just hippie stuff, right?”  P4: “ first responders are really good at saying, oh, that was stupid, you know?” (about a meditative retreat)  “ my wife was a bit of a hurdle because she thought I was gonna get addicted. And I'm a druggie”  “I think taking the stigma barrier is a huge one.”  P5: “There's always a stigma attached to.  This mental health stuff too. I mean, I've noticed, you know, guys kind of avoid me now,”  (Taking about physicians) “They wouldn't sign off for some of the other people in my cohort because they didn't know anything about it. And and they didn't even educate themselves. They just said no.”  P6: “I could see how this stigma behind it could maybe hold back some people”  “The culture can be a thing too, and provide some pressure and like, suck it up mentality”  “even at even around the coffee table and lunch table, more and more guys that I work with are like talking about it and being open … I don't think that was happening at all years ago” | Overall, these quotes convey that the participants struggle with the closed-minded views of others around both Ketamine and their mental health diagnosis. This stigma is described frequently as a barrier to discussing these matters either with their family or peers. Consequently, it is also associated with a lack of support from some people throughout the KAT process. Participants described the culture around mental health for firefighters as not taking mental health seriously, while noting a recent shift away from this mentality. Participants also pointed a lack of media attention on mental health issues that could help raise awareness.  Addressing the research question: stigma is painted as a barrier to openly discussing KAT and to a lesser extent accessing it all together. None of the participants cited stigma as being a deterrent in their decision to do KAT but many mentioned that it may be a barrier for others. Some participants also discussed feeling a need to hide their struggles and need for treatment due to how people around them and society view PTSD/depression and psychedelic treatment. |
| Self-advocacy & self-education | Taking the initiative to inform oneself about trauma and treatments. Making informed and assertive requests to WorkSafe case managers and physicians. | P1: “So I turned to education around trauma and how the brain works and why I was acting the way I was. It sort of was my way of self-therapy.”  P2: “I had already been involved in learning about psychedelics for a number of years.”  “he was really hesitant.  And I actually asked him what he needed and if it would help if he spoke with the medical director”  “I knew what I wanted to do and if she would have signed off, I just would have gone somewhere else.”  “I was lucky that I was prepared to do the paperwork and even knowing, even with all the information I had, it was hard.  It was draining and it was scary.”  “because of my background already I had quite a bit of.I already knew how to navigate that, so yeah.”  P3: “So basically, too bad.  Suck it up .Move on so. At that point, it kind of didn't know where to go, so I just kind of looked for my own help.”  “I would say for anyone that would that is working at this program or thinking about it. Is just to contact people that have gone through it and done it and just like get as much information as you can.”  P4: “He was kind of questioning at why do I need that?... I kind of pushed it and and so I didn't have to push very hard but but he's like yeah, let’s do it.” | Many quotes show patients’ willingness to overcome administrative burdens.  Many either have backgrounds in mental health or have sought out knowledge in order to help themselves.  Many quotes convey a “not-taking-no-for-an-answer” menatilty. Patients often needed to be pushy in order to gain support from physicians.Gaining an adequate understanding in their respective mental health disorders gave them the knowledge to be able to advocate for themselves and navigate difficult situations in front of their physicians and case managers.  Addressing the research question: Accessing KAT requires a lot of self-advocacy. This is needed to overcome barriers such as high amounts of paperwork and hesitant physicians. |
| Awareness about KAT | Disconnect between information about KAT as a treatment option and people who would benefit from accessing it | P2: “There has to be media about it…  Often, people that are struggling with with PTSD or haven't identified that they have it, they wouldn't think to look at this program”  “I don't know that the right information is getting to the people that need to access it.”  P3: “I think just it's one of those things that we that we need to get the word out that you know if the traditional treatment methods aren't helping aren't working then we really need to look at this”  “ I think there needs to be a lot more talk about it amongst you know amongst our peers.”  “I didn't think I was eligible.”  “I think the biggest thing is making everybody aware of it.”  P4: “I didn't talk to them about the ketamine assisted therapy. I didn't know that at the time”  “I never would have thought of it, and until you know, you just reading about it and and magazines and online and that sort of thing”  P5: “There's probably there's a bunch of guys that, that work that probably could use some therapy, but they don't, right. They just drink it away”  P6: “ not many people know about it.  So to get the word out there that this is available in whichever way is possible” | Many quotes point out that many people are not aware that KAT exists or is effective. Participants recommend getting the word out, increasing media outreach and increasing conversations among firefighters as many felt their colleagues also need such treatment.  Addressing the research question: The overarching message is that information about KAT is not being disseminated in a way that reaches those who would benefit from it. Without getting the necessary information, these patients would not have even thought about attempting KAT. |
| Feeling stuck with previous treatment | Other types of therapy (talk therapy, EMDR, CBT, etc) having limited efficacy or short-term effects. | P1: “I actually I was seen a couple different counselors, but just that it just I wasn't getting anywhere.”  “I was stuck on a bit of a hamster wheel and and and I just and so I thought, you know what I need to do more than what I'm doing and I've so let's give it a let's give it a try.”  P2: “ I had tried talk therapy…she did EMT… she did EMDR..I felt better for short periods of time, right? For a little short stints”  “[KAT] could finally help me get through and let go of this trauma and not not be stuck”  P3: “ I was still stuck in the same place”  “we were trying to work through and some of the things were extremely difficult and not really getting a lot of progress”  P4 “ it was a bunch of random counselors that were just, you know, like, oh, I got. I got my counselor. Whatever certificate, and so you know. Yeah, it was the most uncomfortable, probably 35 minutes of my entire life”  P5: (talk therapy) “I mean, things aren't cured by any means, but I think things have gotten better.”  P6: “I’ve done CBT, EMDR, I've seen multiple counsellors. That didn't really work” | A common experience includes feeling stuck and lack of success with other treatments. There were periods where patients felt better, but it was almost cyclic where they had to keep getting the treatment in order to feel better, rather than finding a long term solution.  Research question: Participants try several forms of therapy before resorting to KAT. They opt for KAT when many other modalities have failed to give them a long term solution to alleviate their mental health issues. |
| Logistical barriers | Obstacles to accessing KAT include a lack of financial resources, difficulties getting time off and getting to and from the sessions | P1: “I found because I had to leave the night before in order to make it there on time. So there's a hotel room that I had to pay for and ferry costs and stuff like that,”  (about their department) “ I don't know why they wouldn't just like pay for it and support them while they're off and say we're gonna give you this 12 weeks or 14 weeks or whatever it it we need to have you go through this and take the time that you need and we'll cover the cost for you.”  “it is quite a ways away from the ferry terminal, so it's like a $50.00 or $60.00 cab ride and then it doesn't match up with the ferries either”*  “ I got to wait in the ferry terminal order like 2 1/2 hours before I hop on an hour and a half ferry back to Horseshoe Bay and the whole time I'm coming down from ketamine.”*  P2: “ I would not have been able to do it financially if it wasn't covered by WCB.”  P3: “So on that was probably the biggest barrier is just being away from home in a different place.”  P4 “The time was a thing… I just took the sick time. Before, I would have been afraid to.”  “ it would have been a significant hurdle to (fund) it on our own.”  “I think it's about 6500 bucks, which is pretty significant for three sessions”  “ it's a long day to get from Kelowna to Powell River. And then there's a long trip to get back again, so that was probably the the biggest barrier.”  P5 “I can afford to throw that money at it if I need to. But you know, some other people can't….it cost the hotel cost, the ferries, you know it adds up after a while” | Participants expressed that transportation to the treatment and the cost of treatment are significant barriers. They pointed out that aside from the price of the treatment itself, paying for a hotel and ferries adds financial strain. The burden of traveling for the treatment is further cited as an obstacle as participants struggled with being away from home, the length of time on the road, and coming down from the session while traveling.  Many pointed out that the could not afford the treatment without coverage by departments or unions, but transportation and hotel costs began to add up sigfnicantly. |
| Reaching a breaking point with mental health struggles | Long-term, compounding trauma leading to a crisis or severe effects on a patient's day-to-day life | P1: “ I didn't really have a I didn't really give myself much of a choice.  I had to do something.” (catalyst)  “You continually are always going to other trauma calls, so it kind of builds up layer laughter layer after layer.” (compound)  P2:  “If I want to call it a kind of an existential crisis thing that happened to me. And that's when I was like, OK, something's going on here. Something's wrong.” (breaking pt))  P3: “ well past my burnout date” (compound)  “ I started getting increasingly worried more and more about doing that one call that would be a career-ender” (compound)  P4: “ I don't feel feelings that I feel like I should feel…really easy at shutting off and it's almost impossible to turn them on” (severe side effects)  P5: “Things just kind of went sideways for me after that. I started. Yeah. Night terrors and all sorts of crazy things.” (severe side effects)  P6: “I was in a place of, like, extreme anxiety. It was kind of taking control of me and my life. I wasn't able to work through it anymore” (severe)  “ I had found ways to either ignore it, suppress it, cope, and I was at the point where those weren't working anymore” (breaking point)  “ I just needed to see change, I'd say like I was at a bottom with my whole mental health”  “it was, yeah, a pretty, pretty wild call.  So after I was, I was like instantly like I'm not like I I can't. I can't keep going like this without like at least some help from from somebody.” (breaking point)  “I have a lack of good emotions.I have a significant amount of poor emotions, anger and depression, and that's not who I used to be. | A common sentiment expressed among participants was that they couldn’t keep coping anymore. Many reported having a realization at a particular moment in their life that they had to try something different and there were several mentions of severe symptoms that controlled the participant's life and began to impact their daily functioning.  Addressing the research question: Firefighters seem to be turning to KAT after trauma has affected their lives for long periods. Often the decision is triggered by a crisis or a feeling of reaching rock bottom. Many struggled with prolonged trauma-related symptoms over a period of many years. |
